# Analysis of the current status and associated factors of career calling among novice nurses: a latent profile analysis

**DOI:** 10.3389/fpsyg.2026.1651190

**Published:** 2026-03-04

**Authors:** Dinuo Xin, Dina Xin, Ying Wang, Wenjuan Zhu, Wanling Li, Yuanwei Heng

**Affiliations:** 1 Department of Nursing, Shanxi Bethune Hospital, Shanxi Academy of Medical Sciences, Third Hospital of Shanxi Medical University, Tongji Shanxi Hospital, Taiyuan, Shanxi, China; 2 Third Hospital of Shanxi Medical University, Shanxi Bethune Hospital, Shanxi Academy of Medical Sciences, Tongji Shanxi Hospital, Taiyuan, Shanxi, China; 3 Department of Nursing, Tongji Hospital, Tongji Medical College, Huazhong University of Science and Technology, Wuhan, Hubei, China

**Keywords:** associated factors, career calling, cross-sectional studies, latent profile analysis, novice nurses

## Abstract

**Objectives:**

This study aimed to investigate the current status of career calling among novice nurses, to identify potential subtypes and their population characteristics, and to further explore the factors associated with the different subtypes.

**Methods:**

A cross-sectional descriptive study was used. From January to February 2024, 845 novice nurses from 11 hospitals in Shanxi Province were selected for an online questionnaire survey using convenience sampling. The demographic questionnaire, transition shock of newly graduated nurses scale, medical staff resilience scale, and career calling scale were used as study instruments. Latent profile analysis (LPA) was used to explore the subtypes of novice nurses’ career calling, and multifactorial logistic regression was used to analyze the related factors of novice nurses’ career calling.

**Results:**

Three subtypes of career calling of novice nurses in this study were identified, namely, lacking-calling group (10.3%), stable-calling group (50.0%), and sufficient-calling group (39.7%). Education, weekly working hours, weekly frequency of night shifts, reasons for choosing nursing, level of transition shock, and level of resilience were significantly associated with the three latent profiles of career calling of novice nurses in this study.

**Conclusion:**

Novice nurses’ career calling presents 3 latent profiles and is heterogeneous in this study. Nursing administrators could pay attention to the differences in the level of career calling of novice nurses and adopt targeted management strategies based on the type of characteristics of the population in order to improve the level of career calling of novice nurses, help them develop their careers, and stabilize the nursing workforce.

## 1 Introduction

According to data reported by the World Health Organization, the shortage of nurses has become a serious challenge for healthcare facilities worldwide (World Health Organization, 2024). Novice nurses, as an indispensable reserve for the nursing workforce, are supposed to be an important support to alleviate this predicament. However, statistics on the turnover rate of novice nurses in recent years show a worrying trend (Lyu et al., 2024). A systematic review synthesizing 10 articles showed that the annual turnover rate for novice nurses ranged from 12 to 25% (Bae, 2023). The high turnover rate of novice nurses not only has a potentially negative impact on the quality of clinical nursing care and patient safety but also the stability of the nursing workforce (Li G. et al., 2024). In the face of the growing demand for multilevel and diversified health services, there is an urgent need to increase the attraction and retention of novice nurses in order to effectively alleviate the crisis of nurse shortage. It has been shown that novice nurses’ decisions about whether to join, stay, or leave the profession are influenced by their perceptions, attitudes, and emotions toward the nursing profession (Jiang et al., 2023). In recent years, with the development of positive psychology, there has been increasing evidence of a strong association between career calling and positive personal outcomes. For novice nurses, the transition period is a critical stage in their career development, and career calling at this time is not only able to comprehensively show their career attitudes but also has an important guiding significance in predicting their turnover behavior (Lim et al., 2024).

Career calling refers to an individual’s real love for the career they are engaged in, which is the core driving force that promotes the individual’s pursuit of career goals, and is also an important psychological basis for determining the tendency of their professional behavior (Li F. et al., 2024). Nurses with career calling have a high sense of responsibility and mission for the health and life of patients, and they are willing to pour endless enthusiasm and energy into their work rather than just seeing it as a means of earning a living (Xie S. J. et al., 2024). Numerous studies have confirmed that career calling is positively associated with nurses’ career commitment, professional identity, and job satisfaction, while being inversely related to their intention to leave the profession, which is a key component of their stable development in the nursing profession (Jin et al., 2023; Li C. et al., 2024; Chen and Zhang, 2023). For novice nurses, they are at the beginning of their careers, facing the dual challenges of role transition and environmental change. During this critical period, career calling becomes an important buffer for them to avoid burnout and intention to leave, and plays a central role in securing their continued engagement in nursing in the future (Xin et al., 2024). Therefore, as the new force of the nursing team, novice nurses should cultivate and adhere to a strong sense of career calling, which will enable them to comprehend the significance of their work and affirm the value of their work, so that they can clearly define their own career goals and directions, and pursue and realize their personal career growth and development with a stronger intrinsic motivation (Su et al., 2023).

Career construction theory holds that individual career development is a dynamic process of pursuing mutual adaptation between the subjective self and the external environment, as well as the process of constructing one’s own career development through positive career behaviors and work experiences (Wang and Li, 2024). The ultimate goal of this process is to promote the integration of the person and the environment, so as to successfully adapt to the new work environment (Jilong et al., 2024). According to the career construct theory, career calling is regarded as a dynamic psychosocial resource that may be influenced by both individual traits and external environmental factors at all stages of career development (Wang et al., 2023). Therefore, we will use this theory as a guide to comprehensively explore the relevant factors of novice nurses’ career calling from both individual traits and external environment perspectives.

Transition shock refers to a series of negative emotions such as role maladjustment, anxiety, and self-doubt when novice nurses enter the workplace for the first time due to the complexity and unfamiliarity of the work environment, the intensity of the clinical work, and the mismatch between the actual ability and the expected performance (Ma et al., 2024). Transition shock is associated with novice nurses’ engagement and job satisfaction, and may diminish their enthusiasm and motivation for nursing, which in turn is linked to lower career calling or even consideration of leaving the profession (Kaldal et al., 2023). Previous studies have shown that high levels of transition shock as a primary factor associated with newly graduated nurses’ consideration of leaving within their first year (Wang P. et al., 2024). Therefore, based on career construct theory, we hypothesized that transition shock is an important external factor associated with novice nurses’ career calling.

Resilience is an individual’s ability to recover and self-adjust in the face of adversity, trauma, and stress, and is a positive psychological trait (Dahl et al., 2022). Resilience not only helps individuals adapt to complex and changing environments, but also contributes to individual self-growth and progress, which plays an important role in the transition process of novice nurses. Novice nurses with a high level of resilience can proactively cope with challenges at work, effectively regulate the negative impacts of stress and fatigue, and demonstrate greater autonomy and internal drive (Han et al., 2024). In the process, they find the value and meaning of their work, which in turn is associated with a stronger sense of career calling (Zhang X. et al., 2024). Therefore, based on career construction theory, we hypothesize that resilience is an important intrinsic factor positively associated with novice nurses’ career calling.

For nursing administrators, a prerequisite for achieving targeted support strategies is to accurately delineate the level of novice nurses’ career calling. Currently, most research on career calling among nurses tends to use standardized scale scores to quantify its extent and focuses primarily on exploring correlations among variables. However, this “variable-centered” approach is based on the assumption of population homogeneity, which to some extent ignores inter-individual variability. Given that career calling is a subjective psychological trait that is influenced by individual characteristics and experiences, there are bound to be significant individual differences. In this study, we adopted the “person-centered” method of latent profile analysis (LPA), which can identify different latent subgroups based on individual response patterns on the exogenous variables and observe the characteristics of the subgroups and their heterogeneity, so as to make the results of the study more objective and accurate (Sinha et al., 2021). Therefore, based on the theory of career construction, this study used LPA to explore the differences in the characteristics of career calling among novice nurses and to delve into the relevant factors of different latent subgroups, with the aim of providing a reference for nursing managers to adopt more targeted intervention strategies.

## 2 Methods

### 2.1 Design

This study was a cross-sectional design and followed the STROBE (strengthening the reporting of observational studies in epidemiology) guideline for cross-sectional studies.

### 2.2 Setting and samples

From January to February 2024, novice nurses from 11 tertiary hospitals in Shanxi Province selected for the study using a convenience sampling method. The inclusion criteria were as follows: (a) registered nurses who obtained the professional qualification certificate for nurses; (b) working within 2 years after graduation; and (c) sign the informed consent form and voluntarily participating in this study. The exclusion criteria were as follows: (a) internship nurses; (b) nurses in training; and (c) novice nurses who failed to participate in the survey for various reasons during the survey period. According to Kendall’s sample size estimation criteria, the sample size is 10 times the number of scale entries (Hong et al., 2023). A total of 24 items of Demographic Questionnaire, New Nurse Transition Shock Scale, Medical Staff Resilience Scale, and Nurses’ Career Calling Questionnaire were used in this study. Therefore, the minimum sample size required was 240. Considering the 20% invalid questionnaires, the sample size required was 300.

### 2.3 Ethical considerations

The Medical Ethics Committee of Shanxi Bethune Hospital approved this study (No. YXLL-2023-290). All participants participated in this study voluntarily and could withdraw at any time. In addition, all data collected were used only for this study and were kept confidential and anonymized.

### 2.4 Instruments

#### 2.4.1 The demographic questionnaire

A self-administered demographic questionnaire was used, which focused on age, gender, education, place of birth, one-child status, living environment, marital status, form of employment, length of entry, department, monthly income, weekly working hours, number of night shifts per week, one-way commuting time, and reasons for choosing nursing.

#### 2.4.2 Transition shock of newly graduated nurses scale

This study employed the Transition shock of newly graduated nurses scale developed by Xue et al. (2015) to assess the level of transition shock on novice nurses. The scale contains 4 dimensions and 27 entries, physical (6 entries), psychological (8 entries), knowledge and skills (5 entries), and sociocultural and developmental (8 entries). The scale is based on a 5-point Likert scale ranging from 1 (“does not meet at all”) to 5 (“meets exactly”). The total score ranges from 27 to 135, with higher scores indicating more severe transition shocks. In this study, the Cronbach’s *α* coefficient for the total scale was 0.96, and the Cronbach’s α for the four dimensions were 0.91, 0.91, 0.88, and 0.92, respectively. The content validity index of this scale is 0.906. Confirmatory factor analysis revealed the following parameters: χ^2^ = 956.33, df = 287, χ^2^/df = 3.33, RMSEA = 0.06, CFI = 0.95, NFI = 0.93, IFI = 0.95, TLI = 0.94. These results indicate that the scale possesses good construct validity.

#### 2.4.3 Medical staff resilience scale

We measured novice nurses’ resilience by using the scale developed by Zhu et al. (2016). The scale contains 4 dimensions and 18 entries, namely Decision Coping (6 entries), Interpersonal Connection (4 entries), Rational Thinking (4 entries), and Flexible Self-Adaptation (4 entries). All entries were scored using a 5-point Likert scale ranging from 1 (“completely inconsistent”) to 5 (“completely consistent”), with a total score ranging from 18 to 90, with higher scores indicating higher levels of healthcare staff coping with adversity. The Cronbach’s *α* coefficient for this scale in this study was 0.94, and the Cronbach’s α coefficient for the four dimensions were 0.92, 0.92, 0.90, and 0.84, respectively. Confirmatory factor analysis found that the scale had a good model fit: x^2^ = 211.92, df = 125, x^2^/df = 1.69, RMSEA = 0.06, SRMR = 0.03, CFI = 0.92, TLI = 0.91.

#### 2.4.4 Career calling scale

The career calling of novice nurses was assessed using career calling scale. It was developed by Dobrow and Tosti-Kharas (2011) in 2011 and translated by Pei and Zhao (2015) in 2015. The scale consists of one dimension with 12 entries. The scale is scored on a Likert 7-point scale ranging from 1 (“totally disagree”) to 7 (“totally agree”), with a total score of 12–84, with higher scores indicating higher levels of individual career calling. The Cronbach’s α coefficient for this scale in this study was 0.96. Confirmatory factor analysis revealed the following fit indices for the scale: *x*^2^ = 157.48, RMSEA = 0.08, SRMR = 0.04, NFI = 0.95, TLI = 0.94, CFI = 0.96.

### 2.5 Data collection

Data were collected through the Questionnaire Star online platform. The link to the questionnaire was sent to the novice nurses for completion after obtaining the consent of the director of the nursing department of the surveyed hospital. The beginning of the questionnaire explained the purpose and significance of the study, the notes for completion, and the main content of the questionnaire. Participants were also informed that the study was anonymous and voluntary. To ensure the validity of the questionnaire completion, each question was mandatory and could only be completed once for an IP address. Data verification and entry were carried out by two researchers. Finally, 928 questionnaires were returned. After excluding the invalid questionnaires with obvious errors in logic, strong regularity, and too long or too short response time, 845 replies were valid, and the effective response rate of 91.1%.

### 2.6 Data analysis

Mplus 8.3 software was used to conduct LPA on career calling of novice nurses in this study, using the 12-entry score of the Career Calling Scale as indicators defining the latent profiles, starting with an initial model of 1 category and gradually increasing the number of model categories, and determining the optimal model by conducting a fitness test according to the following three latent profile model evaluation indicators. (1) Akaike information criteria (AIC), Bayesian information criteria (BIC), and the sample size adjusted Bayesian information criterion (aBIC), the smaller the value represents the better the model fitting effect; (2) Entropy is an evaluation index of model classification accuracy, with the value of 0 ~ 1, and the closer to 1, the higher the accuracy; (3) Lo–Mendell–Rubin likelihood ratio test (LMR) and Bootstrapped Likelihood Ratio Test (BLRT) were used to validate the differences in model fit, *p* < 0.05 indicates that the model with k categories is superior to the model with k-1 categories (Nylund et al., 2008). In addition, it is necessary to determine the final number of categories combined with the practical significance of the categorization.

SPSS 26.0 software was used for statistical analysis. Measurement data conforming to normal distribution were expressed as mean ± standard deviation, and intergroup comparisons were made by variance analysis; enumeration data were expressed as number of cases and percentage, and intergroup comparisons were made by chi-square test or rank-sum test. Logistic regression analysis was performed with the categorical results of LPA as the dependent variable and the variables that were statistically significant in the one-way analysis of variance as the independent variables, with *p* < 0.05 indicating that the difference was statistically significant.

## 3 Results

### 3.1 Demographic characteristics of participants

The detailed characteristics of the participants are shown in Table 1.

**Table 1:** Demographic and characteristics by latent profile.

Variable	*n* (%)	Lacking-calling group (*n* = 86)	Stable-calling group (*n* = 423)	Sufficient-calling group (*n* = 336)	χ^2^/F	*P*
Age (years)					3.64	0.457
<23	105 (12.4)	12 (14%)	51 (12.1%)	42 (12.5%)		
23 ~ 25	545 (64.5)	48 (55.8%)	280 (66.2%)	217 (64.6%)		
>25	195 (23.1)	26 (30.2%)	92 (21.7%)	77 (22.9%)		
Gender					6.74	0.034
Male	85 (10.1)	4 (4.7%)	53 (12.5%)	28 (8.3%)		
Female	760 (89.9)	82 (95.3%)	370 (87.5%)	308 (91.7%)		
Education					30.84	<0.001
Junior college	179 (21.1)	12 (14%)	74 (17.5%)	93 (27.7%)		
Bachelor	635 (75.1)	64 (74.4%)	335 (79.2%)	236 (70.2%)		
Master or above	31 (3.7)	10 (11.6%)	14 (3.3%)	7 (2.1%)		
Birthplace					3.80	0.15
Urban	596 (70.5)	53 (61.6%)	305 (72.1%)	238 (70.8%)		
Rural	249 (29.5)	33 (38.4%)	118 (27.9%)	98 (29.2%)		
Only-child status					5.49	0.064
Yes	137 (16.2)	21 (24.4%)	69 (16.3%)	47 (14%)		
No	308 (83.8)	65 (75.6%)	354 (83.7%)	289 (86%)		
Living environment					5.87	0.209
Living with families	366 (43.3)	31 (36%)	189 (44.7%)	146 (43.5%)		
Co-renting	254 (30.1)	27 (31.4%)	116 (27.4%)	111 (33%)		
Live alone	225 (26.6)	28 (32.6%)	118 (27.9%)	79 (23.5%)		
Marital status					0.09	0.954
Married	86 (10.2)	8 (9.3%)	43 (10.2%)	35 (10.4%)		
Unmarried	759 (89.8)	78 (90.7%)	380 (89.8%)	301 (89.6%)		
Employment form					4.07	0.397
Contact system	743 (87.9)	74 (86%)	373 (88.2%)	296 (88.1%)		
Permanent	37 (4.4)	3 (3.5%)	15 (3.5%)	19 (5.7%)		
Other	65 (7.7)	9 (10.5%)	35 (8.3%)	21 (6.3%)		
Duration of work experience (months)					6.33	0.387
1 ~ 6	288 (34.1)	33 (38.4%)	131 (31%)	124 (36.9%)		
7 ~ 12	179 (21.1)	17 (19.8%)	90 (21.3%)	72 (21.4%)		
13 ~ 18	278 (32.9)	27 (31.4%)	154 (36.4%)	97 (28.9%)		
19 ~ 24	100 (11.8)	9 (10.5%)	48 (11.3%)	43 (12.8%)		
Work department					16.99	0.386
Internal medicine	247 (29.2)	25 (29.1%)	123 (29.1%)	99 (29.5%)		
Surgical	150 (17.8)	11 (12.8%)	80 (18.9%)	59 (17.6%)		
Gynecology and obstetrics	44 (5.2)	3 (3.5%)	20 (4.7%)	21 (6.3%)		
Pediatric	51 (6)	8 (9.3%)	24 (5.7%)	19 (5.7%)		
Oncology	27 (3.2)	3 (3.5%)	16 (3.8%)	8 (2.4%)		
ICU or emergency	132 (15.6)	17 (19.8%)	75 (17.7%)	40 (11.9%)		
Operating room	27 (3.2)	3 (3.5%)	11 (2.6%)	13 (3.9%)		
Nursing department	17 (2)	3 (3.5%)	5 (1.2%)	9 (2.7%)		
Other	150 (17.8)	13 (15.1%)	69 (16.3%)	68 (20.2%)		
Monthly income (CNY)					4.95	0.293
≤3,000	366 (43.3)	33 (38.4%)	174 (41.1%)	159 (47.3%)		
3,001 ~ 5,000	409 (48.4)	45 (52.3%)	209 (49.4%)	155 (46.1%)		
>5,000	70 (8.3)	8 (9.3%)	40 (9.5%)	22 (6.5%)		
Weekly working hours					62.92	<0.001
≤40	170 (20.1)	10 (11.6%)	61 (14.4%)	99 (29.5%)		
41 ~ 50	480 (56.8)	43 (50%)	259 (61.2%)	178 (53%)		
51 ~ 60 h	149 (17.6)	17 (19.8%)	86 (20.3%)	46 (13.7%)		
>60 h	46 (5.4)	16 (18.6%)	17 (4%)	13 (3.9%)		
Weekly frequency of night shifts					42.42	<0.001
None	265 (31.4)	20 (23.3%)	116 (27.4%)	129 (38.4%)		
One time	167 (19.8)	12 (14%)	91 (21.5%)	64 (19%)		
2 times	353 (41.8)	36 (41.9%)	185 (43.7%)	132 (39.3%)		
≥3 times	60 (7.1)	18 (20.9%)	31 (7.3%)	11 (3.3%)		
One-way commute length					7.83	0.251
≤10 min	161 (19.1)	15 (17.4%)	78 (18.4%)	68 (20.2%)		
11 ~ 20 min	431 (51)	38 (44.2%)	229 (54.1%)	164 (48.8%)		
21 ~ 30 min	162 (19.2)	23 (26.7%)	78 (18.4%)	61 (18.2%)		
>30 min	91 (10.8)	10 (11.6%)	38 (9%)	43 (12.8%)		
Reasons for choosing nursing					82.59	<0.001
Easily employable	319 (37.8)	33 (38.4%)	169 (40%)	117 (34.8)		
Personal interest	158 (18.7)	2 (2.3%)	50 (11.8%)	106 (31.5%)		
Family wishes	84 (9.9)	10 (11.6%)	52 (12.3%)	22 (6.5%)		
Limited by college entrance examination score	58 (6.9)	12 (14%)	30 (7.1%)	16 (4.8%)		
Specialty transfer	56 (6.6)	11 (12.8%)	35 (8.3%)	10 (3%)		
Other	170 (20.1)	18 (20.9%)	87 (20.6%)	65 (19.3%)		
Transition shock		95.17 ± 20.29	84.16 ± 18.90	69.42 ± 21.64	79.21	<0.001
Resilience		58.11 ± 13.80	66.81 ± 8.57	76.67 ± 8.38	186.85	<0.001

*n* = 845. CNY, Chinese Yuan; F = F test; χ^2^ = chi-square test.

### 3.2 LPA results of career calling in novice nurses in this study

As can be seen in Table 2, when the number of model fits was from 1 to 5, AIC, BIC, and aBIC gradually decreased, and both LMR and BLRT were statistically significant (*p* < 0.05). When the number of categories was 3, the model had the highest Entropy value. And when the number of categories of the model was 4, the Entropy value decreased. Therefore, combining the indicators and considering the interpretability and simplicity of the final results, 3 latent profiles were identified as the best model.

**Table 2:** Model fit indexes of latent profile analysis (*n* = 845).

Model	*k*	AIC	BIC	aBIC	*P*		Entropy	Category probability (%)
					LMR	BLRT		
1	24	33391.60	33505.35	33429.13				100
2	37	28575.80	28751.16	28633.65	0.001	<0.001	0.94	33.6/66.4
3	50	26183.26	26420.23	26261.44	0.004	<0.001	0.96	10.3/39.7/50.0
4	63	25080.77	25379.35	25179.28	0.003	<0.001	0.94	7.2/36.9/22.5/33.4
5	76	24591.95	24952.14	24710.79	0.007	<0.001	0.94	7.2/21.2/33.4/28.0/10.2

k = free parameter; AIC = Akaike Information Criterion; BIC = Bayesian Information Criteria; aBIC = adjusted Bayesian information criteria; LMR = Lo–Mendell–Rubin likelihood ratio test; BLRT = Bootstrap Likelihood Ratio Test.

### 3.3 Characterization and naming of 3 potential profiles

**Figure 1:**
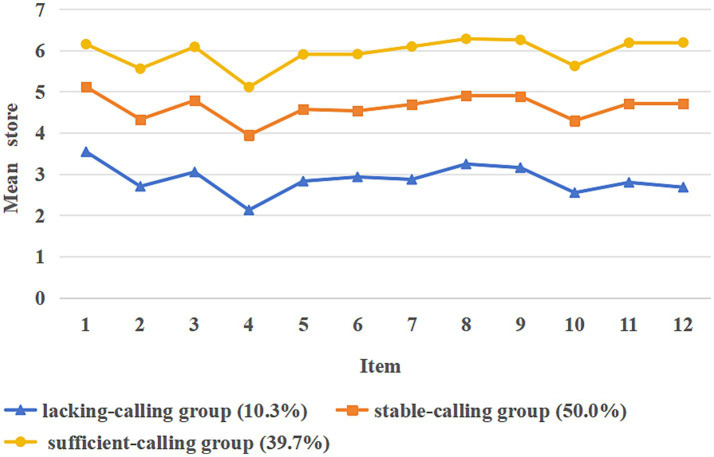
Different profiles of the career calling among new nurse.

Figure 1 shows the scores for each profile on the 12 entries of the novice nurses’ career calling. Three profiles were named based on each latent profile’s score on each entry. Novice nurses in Profile 1 (P1) had low career calling scores, so this profile was named the “lacking-calling group” and accounted for 10.3% of the total; novice nurses in Profile 2 (P2) had mid-level career calling scores, so this profile was named the “stable-calling group” and accounted for 50.0% of the total; and novice nurses in Profile 3 (P3) had high career calling scores, so this profile was named the “sufficient-calling group” and accounted for 39.7% of the total.

### 3.4 Univariate analysis of the factors related to the latent profiles of career calling among novice nurses in this study

The results of the univariate analysis showed statistically significant differences in gender, education, weekly working hours, weekly frequency of night shifts, reasons for choosing nursing, transition shock scores, and resilience scores. The results are shown in Table 1.

### 3.5 Multivariate logistic regression analysis of the factors related to the latent profiles of career calling among novice nurses in this study

Multivariate logistic regression analysis was conducted using the three profiles of novice nurses’ career calling as dependent variables, factors that were statistically significant in the univariate analysis as independent variables, and profile 3 as the reference. The results showed that education, weekly working hours, weekly frequency of night shifts, reasons for choosing nursing, transition shock, and resilience level were the relevant factors of the career calling of novice nurses in this study. The results are shown in Table 3. In our sample, compared with the sufficient-calling group, the probability of being categorized into the lacking-calling group was greater for the master’s degree and above; and novice nurses working ≥ 60 h per week were more likely to be categorized into the lacking-calling group. In addition, novice nurses with ≥ 3night shifts per week were more likely to be assigned to the lacking-calling or stable-calling group. Novice nurses whose reason for choosing nursing was personal interest were more likely to be assigned to the sufficient-calling group.

**Table 3:** The multifactor analysis of career calling of new nurses by logistic regression (*n* = 845).

Variable	Lacking-calling group VS. sufficient-calling group						Stable-calling group VS. sufficient-calling group					
	*β*	SE	Waldχ^2^	*P*	OR	95%CI	*β*	SE	Waldχ^2^	*P*	OR	95%CI
Gender (ref: Female)												
Male	−0.31	0.64	0.24	0.627	0.73	0.21, 2.57	0.58	0.32	3.41	0.065	1.79	0.97, 3.33
Education (ref: Master or above)												
Junior college	−1.92	0.78	6.14	**0.013**	0.15	0.03, 0.67	−0.53	0.57	0.85	0.357	0.59	0.19, 1.81
Bachelor	−1.67	0.70	5.63	**0.018**	0.19	0.05, 0.75	−0.04	0.54	0.00	0.948	0.97	0.33, 2.80
Weekly working hours (ref: >60 h)												
≤40	−1.82	0.69	7.08	**0.008**	0.16	0.04, 0.62	−0.19	0.49	0.16	0.694	0.83	0.32, 2.15
41 ~ 50	−1.49	0.58	6.65	**0.010**	0.23	0.07, 0.70	0.24	0.46	0.29	0.592	1.28	0.52, 3.12
51 ~ 60 h	−1.45	0.65	4.95	**0.026**	0.23	0.07, 0.84	0.39	0.49	0.64	0.424	1.48	0.57, 3.89
Weekly frequency of night shifts (ref: ≥3 times)												
None	−3.21	0.61	27.60	**<0.001**	0.04	0.01, 0.13	−1.39	0.45	9.55	**0.002**	0.25	0.10, 0.60
One time	−2.89	0.65	19.75	**<0.001**	0.06	0.02, 0.20	−0.97	0.46	4.35	**0.037**	0.38	0.15, 0.94
2 times	−2.64	0.57	21.15	**<0.001**	0.07	0.02, 0.22	−1.12	0.44	6.54	**0.011**	0.33	0.14, 0.77
Reasons for choosing nursing work (ref: Other)												
Easily employable	−0.41	0.43	0.90	0.343	0.67	0.29, 1.54	−0.26	0.25	1.12	0.289	0.77	0.48, 1.25
Personal interest	−2.65	0.89	8.85	**0.003**	0.07	0.01, 0.41	−0.92	0.28	10.53	**0.001**	0.40	0.23, 0.70
Family wishes	−0.13	0.59	0.05	0.832	0.88	0.28, 2.81	0.26	0.37	0.50	0.481	1.29	0.63, 2.64
Limited by college entrance examination score	0.21	0.64	0.11	0.742	1.23	0.36, 4.29	0.04	0.43	0.01	0.924	1.04	0.45, 2.40
Specialty transfer	0.93	0.65	2.05	0.152	2.54	0.71, 9.08	0.41	0.45	0.82	0.365	1.51	0.62, 3.65
Transition shock	0.05	0.01	31.90	**<0.001**	1.05	1.03, 1.07	0.02	0.01	19.67	**<0.001**	1.02	1.01, 1.03
Resilience	−0.21	0.02	126.61	**<0.001**	0.81	0.78, 0.84	−0.12	0.01	113.14	**<0.001**	0.89	0.87, 0.91

VS. = versus; SE = standard error; OR = odds ratio; CI = Confidence interval; ref: reference. The bold values indicate statistical significance, with *P* < 0.05.

## 4 Discussion

### 4.1 Latent profile characteristics of career calling among novice nurses in this study

This study was the first to use latent profile analysis to categorize the career calling of novice nurses and to explore the factors associated with the level of career calling of different categories of novice nurses. The results showed that novice nurses’ career calling could be categorized into three latent profiles in this study. In addition, there was significant group heterogeneity in the level of career calling among novice nurses in this study. The results of this study showed that the score of novice nurses’ career calling was (59.58 ± 12.65), which was above the average level, but still lower than the results of the survey of general nurses’ career calling conducted by Fan et al. (2023). This difference may be related to the difference in the average working years of the survey respondents, with most of the nurses in the latter study working for more than 5 years, whereas the subjects in our study all worked for less than 2 years. As a possible explanation, nurses with long working years may have a higher sense of professional value and identity because of their rich working experience and stable working conditions (Zhang C. et al., 2024). Given that novice nurses are the core of the future nursing workforce, the importance of their career calling cannot be ignored. For novice nurses whose career calling is at a low to medium level, nursing managers could strengthen the guidance of their career direction, help them gradually recognize the value of the nursing profession in their work, and stimulate their positive emotions. At the same time, nursing managers need to improve the existing education and training system for nurses, so that novice nurses can combine their personal cognition, professional pursuits and social expectations, deepen their understanding of the value and nature of nursing profession, and thus enhance their sense of career calling (Lu et al., 2025). In addition, career calling is a deep experience that integrates work value, personal pursuit and social responsibility. In order to effectively enhance the sense of career calling of novice nurses, governmental agencies and all sectors of society could give full recognition and respect to the nursing profession, thus accelerating and strengthening the formation and development of nurses’ career calling (Xie L. et al., 2024).

### 4.2 Factors associated with the latent profile of career calling among novice nurses in this study

Logistic regression analysis showed that novice nurses in this study with a master’s degree or above were more likely to be classified in the “lacking-calling group” than those with a junior’s degree or a bachelor’s degree. The reasons for this may be threefold. Firstly, there is a structural contradiction of “focusing on research but not clinical” in China’s postgraduate nursing education, and there is a relative lack of clinical skills training for highly educated novice nurses, coupled with adaptation problems caused by the age factor, could contribute to professional frustration (Zhang C. et al., 2024). Secondly, highly educated novice nurses tend to have a higher expectation of promotion, salary and social status, and when there is a gap between the reality and the expectation, they are prone to have a psychological gap and doubts about their professional value (Chen et al., 2024). Lastly, nursing managers have higher expectations and requirements for highly educated novice nurses, and these nurses may need to take on multiple tasks such as clinical care, research and innovation, and teaching and training at the same time, and this role conflict and work overload may exacerbate burnout (Relster et al., 2023). Based on this, educators in universities could provide more clinical practice courses for graduate students to improve clinical practice skills so that they can better bridge the gap between academia and practice. In addition, nursing administrators could gain insight into the career development needs of novice nurses at all education levels, provide them with personalized career development paths and planning, and offer appropriate career development opportunities, platforms, and resources to meet their self-actualization needs, thereby stimulating and sustaining their sense of career calling (Wangensteen et al., 2018). Finally, nursing managers could strive to balance the research and clinical nursing work of highly educated new nurses, motivate them in their work through performance incentives and other means, so that they can feel their self-worth, thus stimulating a sense of career calling (Zhou et al., 2024).

The results indicated that novice nurses working ≥ 60 h per week were more likely to be classified in the “lacking-calling group” in this study. Previous studies have shown that long working hours for nurses are a major risk factor for endocrine disruption, sleep disorders and other physical dysfunction, and mental health problems (Du et al., 2023). Especially during standardized training, novice nurses often have to work overtime to cope with clinical tasks due to unfamiliar work environments, heavy workloads, and unskilled specialty skills (Tong et al., 2024). In addition, long working hours also encroach on the time they spend on skill acquisition, hobbies, socializing and taking care of their families, which may contribute to work-life imbalance that is associated with reduced quality of life and career well-being, factors that could be inversely linked to the sense of career calling (Kim et al., 2024). Therefore, with full consideration of the workload of the ward, nursing human resources, nurse–patient ratio and other factors, nursing managers could scientifically arrange the shifts according to the individual abilities, strengths and weaknesses of the novice nurses, reasonably distribute their workloads and working hours, and ensure that they have enough time for rest and recovery, so that they can realize the balance of work and life. At the same time, nursing managers could pay special attention to novice nurses who frequently work overtime, explore the root causes of their overtime work in-depth, and provide targeted guidance and support to help them gradually master the rhythm of their work and reduce the frequency of their overtime work, so as to break the vicious circle of a low sense of career calling (Wang X. et al., 2024).

The results indicated that novice nurses with ≥3night shifts per week had a greater probability of being classified in the “lacking-calling group” in this study. Nursing managers often arrange more night shifts for novice nurses because of their age and physical strength advantages. However, frequent night shift work is associated with health problems such as disrupted life rhythm, sleep disorders, and decreased immunity (Boivin et al., 2022). Meanwhile, the high intensity and high risk of night shifts pose a challenge to novice nurses’ autonomous decision-making ability, emergency response ability, and psychological quality, which may contribute to tension, anxiety, and sense of helplessness, potentially affecting their work performance and being linked to burnout (Li L. et al., 2024). In addition, frequent night shifts may also reduce the time novice nurses spend with their families and friends, which may strain their family-social relationships and increase emotional burden, thereby possibly undermining the perceived value of their profession (Al-Hrinat et al., 2024). Therefore, nursing managers could optimize the allocation of human resources, dynamically adjust the frequency of night shifts according to the novice nurses’ own ability and tolerance, and give them a certain degree of scheduling autonomy. At the same time, it is necessary to improve the night shift support system to alleviate physical and mental pressure and improve motivation by guaranteeing rest time and nutritional supply for novice nurses and implementing the night shift performance incentive mechanism (Chen et al., 2023). In addition, clinical instructors could assess the work adaptation status of new nurses, strengthen the training of new nurses’ night shift risk and emergency response ability, and improve their autonomous decision-making and coping ability, so as to better adapt to the work rhythm of night shifts and further stimulate new nurses’ career calling (Wang J. et al., 2024).

The results of indicated that novice nurses in this study whose reason for choosing nursing was personal interest were more likely to be categorized in the “sufficient-calling group.” Research has found that interest and love are often the prerequisites and foundation for success. Interest is an important motivator to stimulate personal potential and a key factor to promote continuous learning and improvement of individuals (Zhang et al., 2023). Therefore, those novice nurses who chose the nursing specialty based on personal interest tended to have a deeper understanding of the disciplinary development and prospects of the nursing specialty and a higher sense of professional identity, and were able to appreciate the mission and value of the nursing profession more deeply. On the contrary, those new nurses who chose the nursing specialty for other reasons may be more confused in their career planning and have difficulty in finding their own value from their work, which could correspond to lower career benefit and calling (Özaras et al., 2024). Therefore, nursing educators could guide novice nurses to establish correct professional values and deepen nursing professional value education in the school education and clinical practice stage. At the same time, nursing administrators could identify early on novice nurses who do not choose the nursing profession out of personal interest, and take the initiative to assist them in formulating career plans, clarifying career goals, and exploring points of interest in their careers. In addition, external factors such as appropriate incentives can be used to stimulate the internal motivation of novice nurses and to recognize their professional value, thus stimulating their strong interest and enthusiasm for nursing.

The results of the regression analysis showed that novice nurses with higher transition shocks had lower career calling in this study. When novice nurses transition from school to hospital, they face various challenges such as environment and role change, work pressure, and interpersonal relationships, which are linked to multiple levels of transition shock. According to the conservation of resources theory, high levels of transition shock may act as a stressor that could deplete the physical and psychological resources of novice nurses. This depletion might not only be associated with reduced motivation for learning and work but could also contribute to doubts regarding the meaning and value of the nursing, which in turn may be related to a weaker sense of career calling (See et al., 2023). In this regard, nursing managers could consider adopting a systematic support strategy. First, enhancing the pre-service training system through standardized process training and departmental rotation guidance may help novice nurses quickly master work skills and reduce the pressure of the adaptation period. Secondly, establishing a “one-to-one” mentorship system, in which experienced nurses can provide practical clinical guidance and transfer professional values, could strengthen the professional self-confidence and professional identity of novice nurses. Finally, a dynamic tracking mechanism is implemented to regularly assess the work status and psychological stress level of novice nurses, and provide timely targeted support when adaptation barriers occur, so as to ensure the successful completion of the transition of professional roles and further deepen the sense of career calling (Kim and Yeo, 2021).

The results of the regression analysis showed that novice nurses with higher resilience scores were more likely to be categorized into the “sufficient-calling group” in this study, which is consistent with Sun’s findings (Sun et al., 2022). Resilience, as a positive psychological resource, is a protective factor for individual mental health (Xing et al., 2024). When facing busy and complex work environments and stressful events, novice nurses with high resilience are able to actively and effectively manage stress and obstacles at work, alleviate the negative effects of stress, and experience more positive emotions. This process of fighting adversity not only gives them a sense of work accomplishment and value, but also further stimulates their enthusiasm for their work, which may in turn be positively associated with career calling (Li Y. R. et al., 2024). Based on this, nursing managers can implement multidimensional intervention strategies. First, novice nurses could be encouraged to strengthen their individual psychological capital and improve their self-adjustment ability under adversity through stress regulation skills training. Secondly, a multi-level social support system could be constructed to establish a three-dimensional linkage mechanism between managers, teams, and colleagues to help them cope with various challenges in their work and life through effective communication mechanisms. In addition, it is recommended to integrate developmental psychological intervention programs and adopt intervention strategies such as group counseling, cognitive-behavioral intervention, and positive thinking stress reduction to systematically improve novice nurses’ stress cognitive assessment ability and emotion regulation strategies, thus enhancing their resilience in professional adversity and supporting the transformation of professional identity to career calling (Zhang Y. et al., 2024).

### 4.3 Limitation

Although this study has certain findings, it also has several limitations. Firstly, the study adopted convenience sampling, and the sample was only from Shanxi Province. This might cause systematic differences between the sample characteristics and the overall characteristics, introducing selection bias; at the same time, regional medical culture and economic levels might also limit the generalizability of the results. Future research can adopt multi-center stratified random sampling, including novice nurses from different provinces and different levels of hospitals, to improve the representativeness of the sample and the universality of the results. Secondly, the cross-sectional design cannot establish causal relationships between variables or the direction of dynamic influences. Further research can use longitudinal tracking to further reveal the evolution trajectory of career calling. Finally, although the scales used have good reliability and validity, self-report measurement still cannot completely avoid common method bias and social expectation effects. In the future, objective indicators or qualitative interviews can be combined to understand the professional experience of novice nurses more comprehensively and deeply, thereby providing more solid and reliable basis for management intervention.

## 5 Conclusion

In this study, three latent subgroups of novice nurses’ career calling were identified through latent profile analysis: “lacking-calling group,” “stable-calling group” and “sufficient-calling group,” and the majority of novice nurses were in the “stable-calling group.” In this study, novice nurses in the different profiles differed in education, weekly working hours, weekly night shifts, reasons for choosing nursing, level of transition shock, and level of resilience. Nursing managers could pay attention to the differences in career calling of novice nurses, identify the categories and population characteristics of novice nurses’ career calling early, and adopt targeted guidance and training to enhance the level of career calling of novice nurses, so as to alleviate the crisis of nursing shortage.

## Data Availability

The original contributions presented in the study are included in the article/supplementary material, further inquiries can be directed to the corresponding author/s.
